# The effect of microbial challenge on the intestinal proteome of broiler chickens

**DOI:** 10.1186/s12953-017-0118-0

**Published:** 2017-05-30

**Authors:** Emily L. O’Reilly, Richard J. Burchmore, Nicholas H. Sparks, P. David Eckersall

**Affiliations:** 1 Institute of Biodiversity, Animal Health and Comparative Medicine, College of Medical, Veterinary and Life Sciences, Glasgow University, Glasgow, UK; 2 Glasgow Polyomics Facility, College of Medical, Veterinary and Life Sciences, Glasgow University, Glasgow, UK; 3 The Roslin Institute and Royal (Dick) School of Veterinary Studies, Easter Bush, Midlothian, EH25 9RG UK

**Keywords:** Intestine, Chicken, Villin, Proteome, Apoptosis

## Abstract

**Background:**

In poultry production intestinal health and function is paramount to achieving efficient feed utilisation and growth. Uncovering the localised molecular mechanisms that occur during the early and important periods of growth that allow birds to grow optimally is important for this species. The exposure of young chicks to used litter from older flocks, containing mixed microbial populations, is a widely utilised model in poultry research. It rarely causes mortality but effects an immunogenic stimulation sufficient enough to cause reduced and uneven growth that is reflective of a challenging growing environment.

**Methods:**

A mixed microbial challenge was delivered as used litter containing *Campylobacter jejuni* and coccidial oocysts to 120 male Ross 308 broiler chicks, randomly divided into two groups: control and challenged. On day 12, 15, 18 and 22 (pre- and 3, 6 and 10 days post-addition of the used litter) the proximal jejunum was recovered from 6 replicates per group and differentially abundant proteins identified between groups and over time using 2D DiGE.

**Results:**

The abundance of cytoskeletal proteins of the chicken small intestinal proteome, particularly actin and actin associated proteins, increased over time in both challenged and control birds. Villin-1, an actin associated anti-apoptotic protein, was reduced in abundance in the challenged birds indicating that many of the changes in cytoskeletal protein abundance in the challenged birds were as a result of an increased rate of apoptosis. A number of heat shock proteins decreased in abundance over time in the intestine and this was more pronounced in the challenged birds.

**Conclusions:**

The small intestinal proteome sampled from 12 to 22 days of age showed considerable developmental change, comparable to other species indicating that many of the changes in protein abundance in the small intestine are conserved among vertebrates. Identifying and distinguishing the changes in proteins abundance and molecular pathways that occur as a result of normal growth from those that occur as a result of a challenging microbial environment is important in this major food producing animal.

## Background

The gastro-intestinal (GI) tract of poultry is an important proteomic target for research. Enclosed by a single layer of polarised epithelial cells affixed to an extracellular matrix known as the basement membrane [1], the GI tract serves as a selective barrier between the tissues of the bird and the luminal environment [2] and provides the mechanisms by which the body derives nutrients from its environment. Its role in nutrient absorption, growth and maintaining the overall health of the bird makes it an apt proteomic target, further analysis of which allows the molecular mechanisms involved in responses to disease, dietary supplementations and feed utilisation to be fully understood. Identifying the mechanisms by which intestinal pathogens have their most deleterious effects will enable a deeper understanding of enteric health in poultry.

Studies of the chicken intestinal proteome have found that proteins in the brush border involved in digestive function, maintenance of membrane potential, membrane trafficking and cytoskeletal organisation change over time from day of hatch to 14 days post hatch, with changes differing between genetic lines [3]. Bacteriocins, a heterogeneous group of proteinaceous compounds lethal to bacteria other than the producing strain, were found to cause differential abundance of metabolic-related proteins within the jejuna, many of which were antioxidants [4]. The feeding of the probiotic *Enterococcus faecium* (*E. faecium*)*,* resulted in the differential abundance of 42 intestinal mucosal proteins, including those related to intestinal structure and immune and antioxidant systems of which 6 were abundant in the broilers fed *E. faecium* [5]. High atmospheric ammonia exposure was found to cause differential abundance of 43 intestinal mucosal proteins, increasing the abundance of proteins related to oxidative phosphorylation and apoptosis and reducing those related to cell structure and growth, transcriptional and translational regulation, immune response, oxidative stress and nutrient metabolism; indicating that exposure to ammonia triggered oxidative stress and interfered with nutrient absorption and immune function in the small intestinal mucosa of broilers [6].

The GI tract microbiota plays an essential role in the growth and health of the bird and as the bird ages the microbial profile becomes more complex [2]. The environment and dietary composition affect the microbiota and because the microbiota affects intestinal development, the mucosal architecture and the mucus composition, the intestine will therefore also be affected by a range of factors associated with the birds environment and management [7]. A healthy microbiota imparts a positive effect on health and wellbeing of an animal, and this effect will be mediated, at least in part, at the intestinal surface, where microbiota and enterocyte meet. The majority of broilers in modern poultry productions systems are reared on litter, a mixture of the initial bedding materials, excreta, feathers, unconsumed feed and other detritus from the chicken [8, 9]. Reuse of litter, widely practiced in the USA and other countries, is known to quantitatively and qualitatively alter the avian gut microbiota through the ingestion of litter borne enteric pathogens thereby affecting the overall health and growth performance of broiler chickens [10]. Used litter increases microbial complexity, acting as an immunological challenge to the chicks and negatively affecting growth rates [8–10]. Exposing chicks to used litter under experimental conditions provides a way of investigating how the intestinal proteome responds to this challenge specifically as well as having the potential to provide more general information on the molecular mechanisms operating within the GI tract. The intestinal microbiota has been shown to effect the intestinal proteome of pigs [11]. When gnotobiotic pigs are mono-associated with *Lactobacillus* (*L.*) *fermentum* or non-pathogenic *Escherichia* (*E.*) *coli,* both commensal bacteria commonly found in the neonatal pig intestine, proteins related to lipid metabolism, proteolysis and apoptosis and plasma proteins were markedly affected. *E. coli* was found to have a profound effect on cell proliferation and enterocyte migration, whereas *L. fermentum* had more of an immunoregulatory role [11]. Human studies have largely focused on the microbiota metaproteome, with a resultant body of evidence linking the microbiota profile to health and disease [12]. Because the intestinal epitheilial barrier plays such a critical role in mediating the relationship between microbiota and host, during both homeostasis and disease, studies have also targeted the host intestinal proteome particularly in relation to the pathogenesis of inflammatory bowel diseases such as Crohn’s disease and colitis [13, 14]. This host-centric approach, focusing on proteins expressed by the host has been widened to include the faecal proteome [15], as the microbial colonisation on the GI tract impacts the pattern and diversity of host proteins in the faeces [16].

The aim of this study was to examine the changes in the proteome of the small intestine of the chicken in response to a microbial challenge over a ten day period. Differential gel electrophoresis (DiGE) was used to study the proteome and the microbial challenge was provided by adding used litter to 6 replicate challenge pens. Detailing the proteomic changes that occur in the intestine in the growing bird and how disruption to the intestinal homeostasis affects the intestine proteome will contribute to the understanding of small intestinal development and response to microbial challenge.

## Method

### Animals and experimental design

The experimental design and procedure was approved by SRUC’s Animal Experiments Committee (AU AE 40–2012) and complied with the conditions of the relevant Home Office licences. At day-old, 120 male Ross 308 broiler chicks were randomly divided into two groups: control and challenged. Birds were divided into 6 replicates per group, housed in floor pens containing 10 birds per pen in an environmentally controlled room (with conditions maintained as appropriate for the age of bird). Chicks were fed a commercial starter ration until 10 days of age after which they were moved onto a grower ration for the remainder of the study. On day 12, litter from a commercial unit housing broilers aged 35 days was recovered from the top layer, manually broken down and 1 kg was added to each of the challenge pens. On day 12, 15, 18 and 22 (3, 6 and 10 days post-addition of the used litter) the chicks in each pen were bulk-weighed and one bird from each pen was randomly selected and following cervical dislocation, the proximal jejunum was recovered, flushed lightly with physiological saline to remove digesta and a 5 cm section removed, frozen immediately and stored at −80 °C till use.

### Bacteriology

An aliquot of the challenge litter taken on the day of collection and day 22 digesta, recovered and pooled from jejunal and ileal regions of the challenge and control groups, were analysed at the Avian Science and Research Centre (ASRC) microbiology laboratory (SRUC, Auchincruive, UK). From these samples 10 g was made to 100 g with the addition of sterile saline and homogenised in a stomacher for 45 seconds to make a 10^−1^ dilution. Further tenfold dilutions were prepared in sterile universal bottles down to 10^−9^. Dilutions were formed in duplicate. For both litter and digesta aerobic and anaerobic total viable counts (TVC) were determined following 48 hr incubation at 37 °C on nutrient agar. *Lactobacillus* spp. and *Campylobacter* spp. were enumerated using Man Rogosa Sharpe agar (MRS, Oxoid CM0361) 1 ml pour overlay plates (both layers identical) incubated microaerophilically for 48 h at 37 °C and charcoal-cefoperazone-deoxycholate agar (CCDA) incubated microaerophilically for 48 h at 41.5 °C respectfully. For *Campylobacter* spp. some confirmatory steps were performed, namely Gram stain and oxidase test. For enumeration of *Clostridium perfringens* in the litter, samples were incubated anaerobically on Tryptose Sulphite Cycloserine agar (TSC, Oxoid UK CM0587) overlay plates (bottom layer perfringes agar base (PAB), TSC and egg yolk/ top layer with PAB and TSC) for 48 h at 37 °C. Digesta coliform counts were enumerated following incubation for 48 h at 37 °C on MacConkey Agar without salt.

### 2D DiGE

The DiGE method described by [17] was used with the following modifications: 0.5 g of jejuna from each chicken was ground a pestle and mortar with liquid nitrogen and pooled to create control and challenge groups for days 12, 15, 18 and 22. For each group 100 mg of the pooled ground jejuna was vortexed with 500 μl of DiGE lysis buffer (7 M urea, 2 M thiourea, 25 mM Tris, 4% CHAPS) and an acetone precipitation was performed and to the final pellet 200 μl of DiGE lysis buffer was added. A Bradford assay determined the protein concentration, and the samples adjusted to 5 mg/ml with DiGE lysis buffer. For each time point 50 μg of intestinal lysate from both challenge and control groups were labelled to both Cy3 and Cy5 DiGE Fluors. A pooled internal standard, containing equal volumes of protein from control and challenged groups at each time point were bound to Cy2 dye. The Cy3 and Cy5 bound samples together with the Cy2 bound standard protein were added to a rehydration buffer (6 M urea, 2 M thiourea, 4% CHAPS, 0.002% (w/v) bromophenol blue) into which 3.5 mg of dithiothreitol (DTT) and 1.75 μl IPG pH 4–7 buffer (GE healthcare biosciences 17-6000-86) was added before being applied to a 24 cm 4–7 IPG strip (GE Healthcare Life Science 17-6002-32), covered with 1 ml of mineral oil and focused to 8000 Volts over 27 h. The IPG strips were washed in 10 mg/ml DTT and 25 mg/ml 2-Iodoacetamide IEF equilibrium buffers for 15 minutes each. For the second dimension the IPG strip was placed onto a 24 cm 12.5% SDS-PAGE gel and ran in a ETTAN DALT system with 2% SDS running buffer at 1 V per gel for 18 h. Gels were scanned on a Typhoon 9400 laser scanner. For spot picking 2D preparatory gels were produced as described for the DiGE by mixing 500 μg of protein with 350 μl of rehydration buffer. Following 2D separation the gels were fixed and stained in colloidal Coomassie for 48 h.

### Image analysis

DiGE images were analysed using DeCyder v7.0 (GE Healthcare, Amersham, UK). A two-way analysis of variance (ANOVA) was performed on the protein log spot abundance derived from the normalized spot volume standardized against the intra-gel standard with a false discovery rate of 1%. Differentially abundant protein spots volumes whose log standardised abundance were statistically significant both between challenged and control groups over the four time points were selected for identification, matched to the preparatory spot maps and excised from the preparatory gels. Spots that had significantly different abundance for a single variable, either time point or challenge were also selected.

### Spot excision and digestion

Selected protein spots were manually excised, diced, washed in 500 μl of 100 mM ammonium bicarbonate for 30 minutes, twice washed with 500 μl of 50% acetonitrile in 100 mM ammonium bicarbonate for 30 minutes and finally washed in 50 μl of 50% acetonitrile for 10 minutes, after which the solvent was removed and the gel pieces dried in a Speed-Vac centrifuge. For in-gel digestion, gel pieces were rehydrated with sequencing grade trypsin (Promega, WI USA, V511A) re-suspended in 20 μl of 25 mM ammonium bicarbonate. Following a further rehydration with 20 μl of 25 mM ammonium bicarbonate, the protein was allowed to digest overnight at 37 °C. Following centrifugation the liquid was transferred to a 96 well plate. To the pelleted gel pieces, 20 μl of 5% formic acid was added and following a 20 minute incubation with agitation 40 μl of acetonitrile was added and the incubation continued for 20 minutes. The samples were centrifuged and the supernatant added to the liquid in the 96 well. The combined extracts were dried down completely in a vacuum centrifuge.

### Protein identification

Proteins were identified using nanoflow HPLC electrospray tandem mass spectrometry (nLC-ESI-MS/MS) as described in [17] with protein identifications assigned using the Mascot search engine to interrogate protein sequences in the NCBI Genbank databases for *Gallus gallus* and bony vertebrates, allowing a mass tolerance of 0.4 Da for both MS and MS/MS analyses.

### Western blot analysis

To 5 μg of gut lysate, 10 μl of Laemelli buffer (BioRad 1610737) was added, heated at 95 °C for 4 minutes and the proteins separated in using a BioRad Criterion XT Bis-Tris precast gel (BioRad #345-0124) over 25 min at 200 V in MOPS running buffer (BioRad #161-0788). The proteins were transferred to nitrocellulose paper (NCP) (Bio-Rad 162–0112) in a Criterion blotter (Bio-Rad 170–4070) with transfer buffer (25.012 mM Tris, 191.82 mM Glycine, Methanol 20% (v/v)) at 70 V over 1 h. The NCP was blocked overnight with 1% (w/v) non-fat milk protein in Tris buffered saline (18 mM Tris, 123.2 mM NaCl) (TBS) and washed three times with 0.1% tween (TBS-T). Incubation with primary antibodies: mouse anti-chicken villin antibody (MCA292 AbD Serotec Kidlington, UK) diluted 1:50,000 with TBS-T and rabbit anti-chicken apolipoprotein AIV (apo AIV) (Ab113108, Abcam, Cambridge, UK), diluted 1:4000 with TBS-T, over 1 h at room temperature was followed with washing (TBS-T) and a 1 h room temperature incubation with donkey anti-mouse IgG conjugated to HRP (Ab6820, Abcam, Cambridge, UK) and goat anti-rabbit IgG conjugated to HRP (Ab6721, Abcam, Cambridge, UK) diluted 1:20,000 with TBS-T; secondary antibodies to the villin and apo AIV primary antibodies respectfully. The NCP was washed with TBS-T and developed with enhanced chemiluminescence (ECL) substrate (32106 ThermoFisher Scientific, Paisley, UK). This was replicated three times at these antibody concentrations.

### Statistical analysis

Mean pen weights, taken over the four time points were analysed using regression analysis on Minitab v.16 [18] and data are expressed as mean ± SD and *p* < 0.05 was considered significant. DeCyder, after spot matching, used two-way ANOVA in the BVA module.

## Results

### The effect of re-used litter on growth

**Fig. 1:**
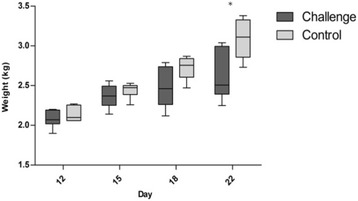
Box plot of pen weights of challenge and control groups from day 12 to day 22, median ± minimum and maximum values (* <0.05)

The used litter, introduced at day 12, resulted in a significantly lower (*P* < 0.05) mean weight for the challenged birds at day 22, when compared to the control, with mean body weights of 374 g (±41 g) and 441 g (±34 g) respectively (Fig. 1). No mortalities were recorded during the period of the trial. A representative sample of the challenge litter was submitted for microbial analysis on the day of collection and *Clostridium perfringens*, *Lactobacillus, Campylobacter jejuni* and *Eimeria* were identified. The digesta from the small intestine was recovered on day 22. The microbial populations for the challenged and control birds were found to have similar numbers of total viable count (TVC) for aerobic and anaerobic bacteria and for coliform bacteria. *C. jejuni* was identified in the used litter and the ileal digesta of challenged birds at 22 days of age, while the control digesta was negative for *C. jejuni*. The *Eimeria* oocyst count from the used litter was 27,000 oocyst per gram which corresponds to lesion score 1 [19]. This oocyst count was within the expected range for a poultry unit at 35 days.

### DiGE

**Fig. 2:**
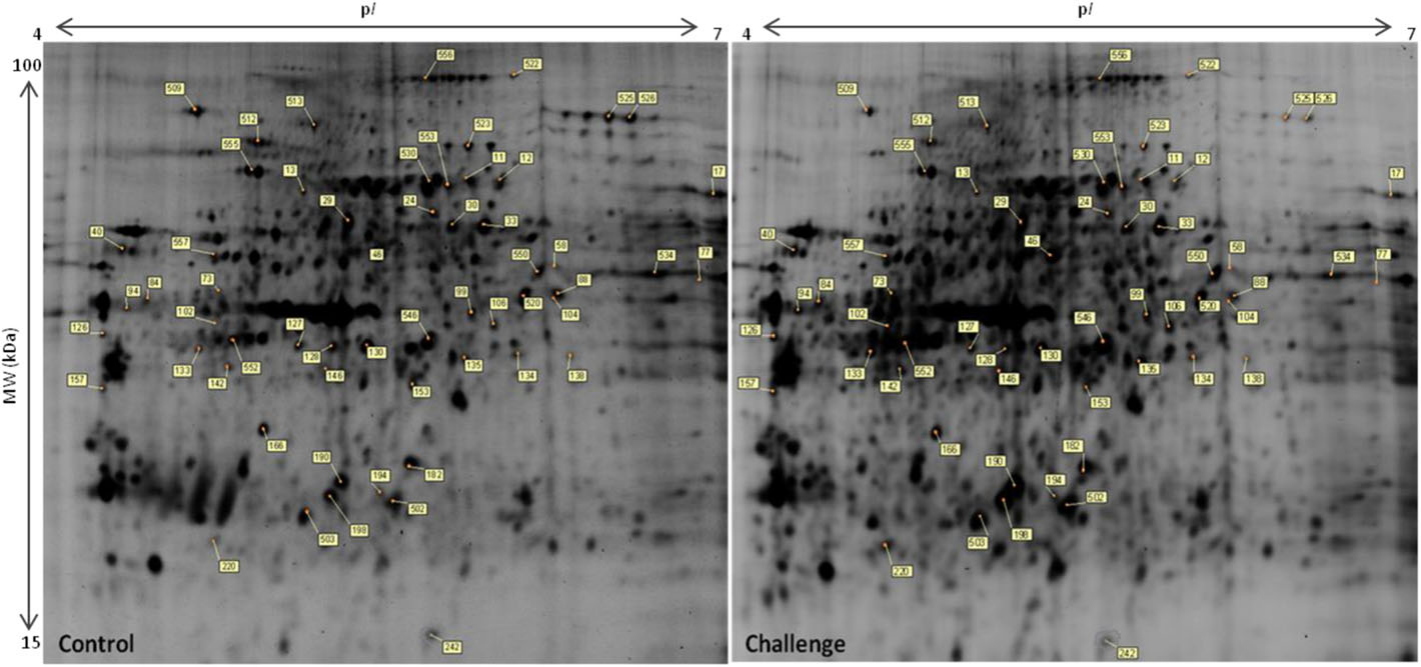
Spot maps from a control and challenged samples from day 22

**Fig. 3:**
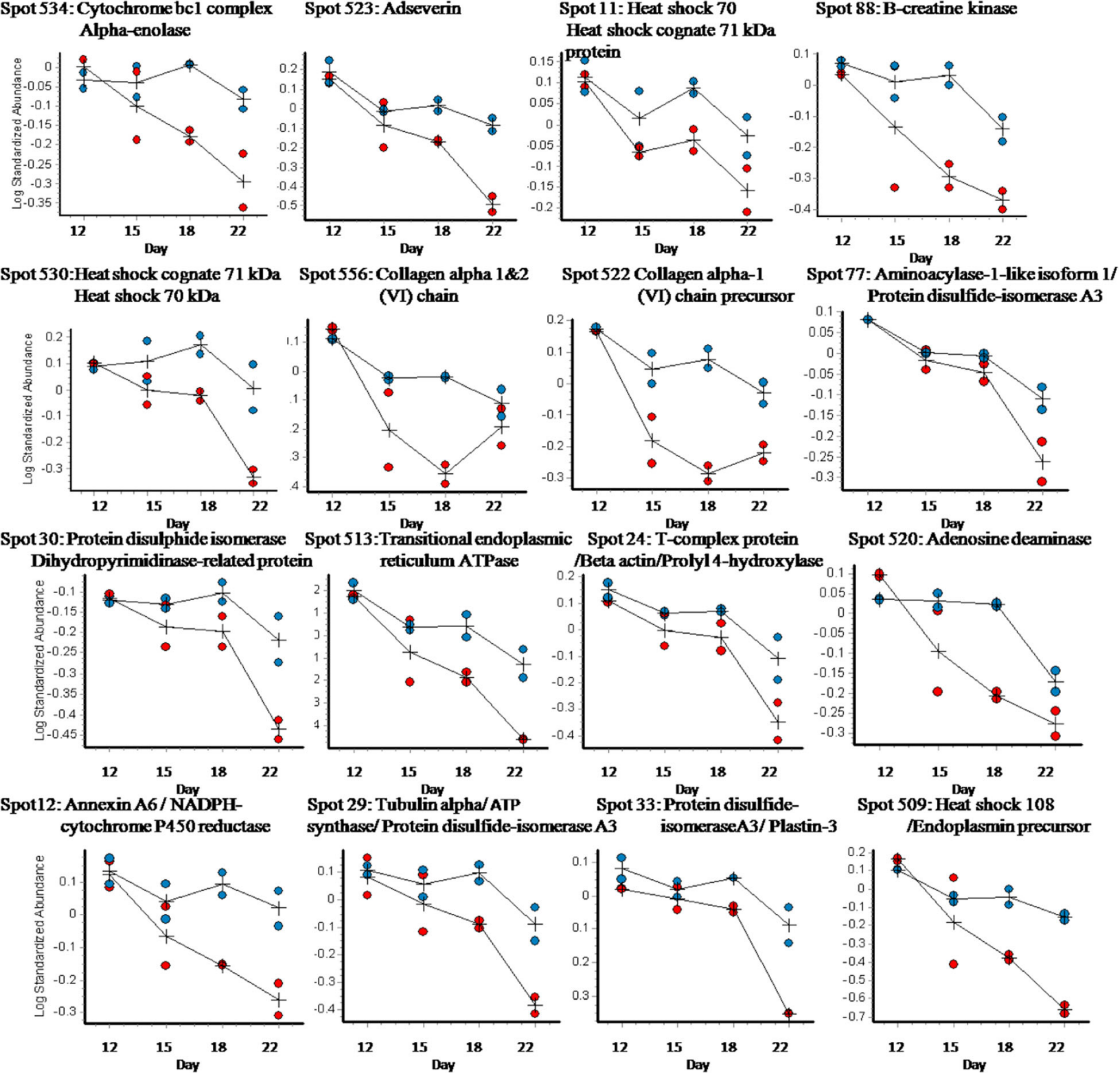
Protein spots that decreased in abundance over time as a result of microbial challenge

**Fig. 4:**
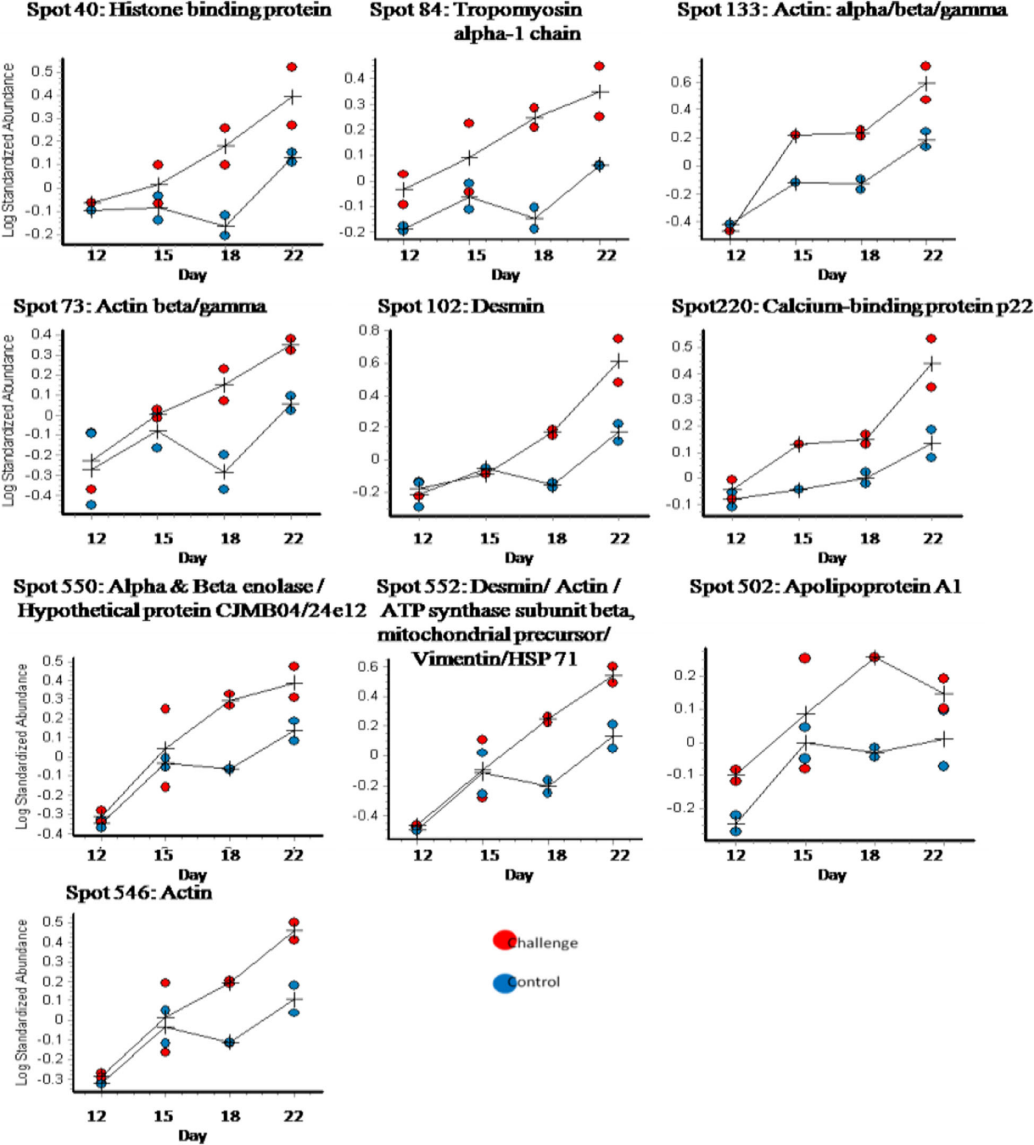
Protein spots that increased in abundance over time as a result of microbial challenge

**Fig. 5:**
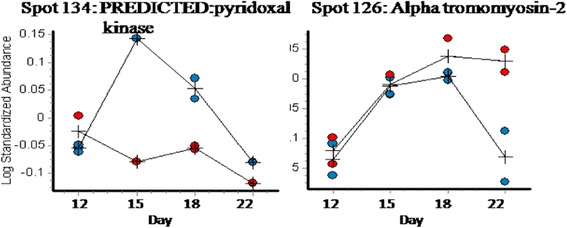
Protein spots that showed significant but varied changes over time as a result of microbial challenge

**Fig. 6:**
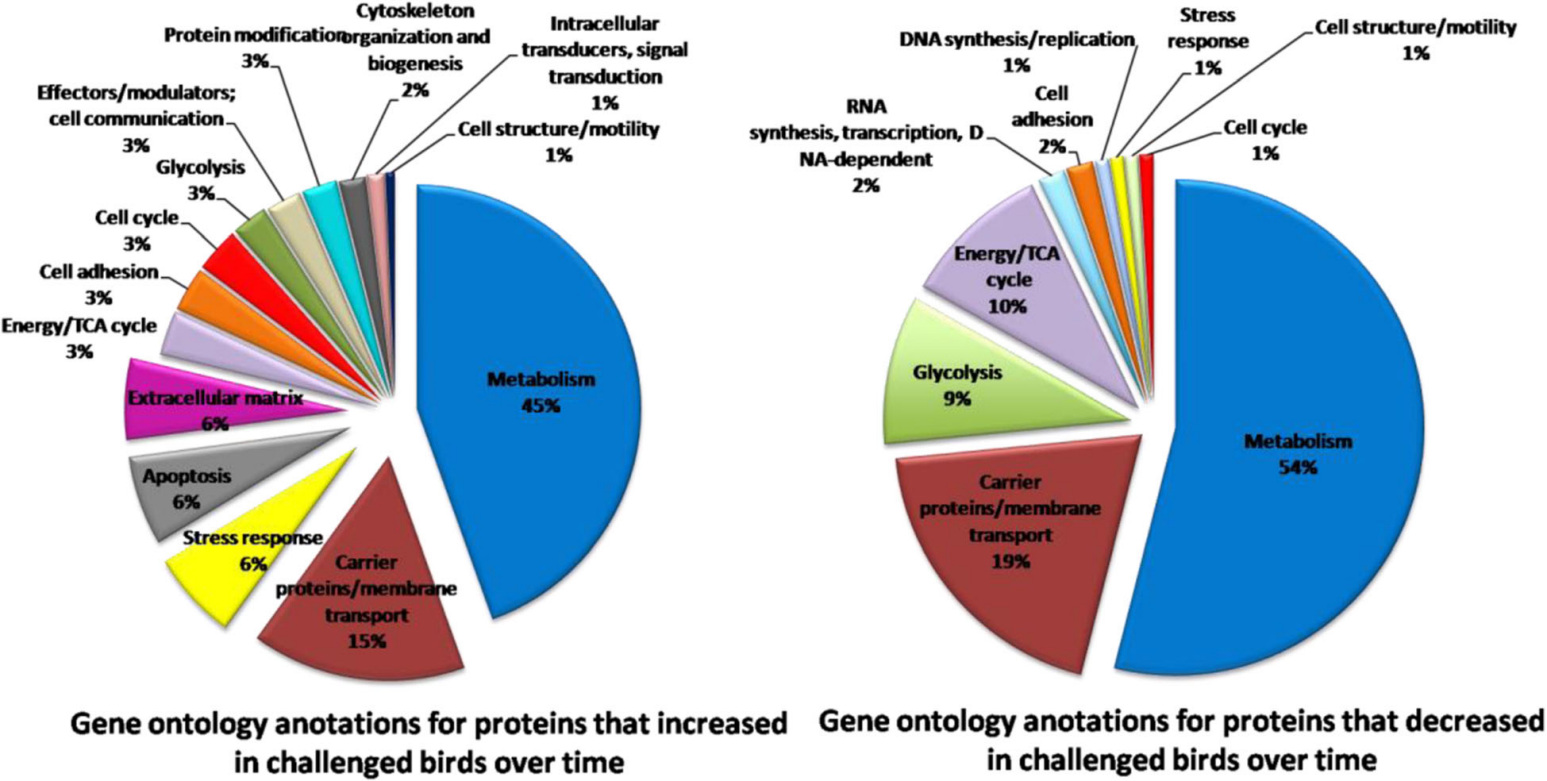
Using gene ontology annotations, the molecular and biological functions of all of the proteins from the spots that increased and decreased as a result of a microbial challenge over time were compared (animalgenome.org/cgi-bin/util/gotreei)

Analysis of the gels using DeCyder reported 792 spots after an area of interest and exclusion parameters were defined. Of these spots, 280 were matched across all gels. A two-way ANOVA identified spots whose abundance changed significantly between challenged and control samples and those which changed abundance significantly over time (days 12 – 22 after hatching). Figure 2 details the spot maps from day 22 challenge and control groups. Of the 792 spots reported, 28 spots were differentially abundant over time as a result of microbial challenge (Table 1). Of these, 10 showed increased abundance in the challenged birds compared to the control birds from day 12, 16 decreased in the challenged birds from day 12. There were 2 spots that showed variable but significant changes between the two groups over the 4 time points. The log standardised abundance of these protein spots were plotted over time for challenged and control groups. Figures 3, 4 and 5 depict the graphs of all 28 spots differentially abundant over time as a result of the microbial challenge. Spots that showed significant differences in abundance for only one of the variables (either litter challenge or time) were also identified (Table 2), 23 spots were differentially abundant over time in all birds and there were 6 spots that showed differential abundance between the control and challenge birds only and did not show significant changes over time. Table 2 lists and details these spots and the proteins they contained. A comparison of molecular and biological functions of the proteins found to increase and decrease as a result of the microbial challenge were compared using the gene ontologies (GO) [20]. The GO terms for all the proteins that were either increased in abundance or decreased in abundance were compared (Fig. 6). Proteins associated with the stress response, apoptosis and the extra-cellular matrix were found increase in abundance in the challenged birds overtime. Proteins associated with metabolism, energy/TCA cycle ad glycolysis and membrane transport were found to reduce in abundance.

**Table 1:** Proteins differentially abundant over time as a result of the microbial challenge. The p-values for both variables: time and litter are given for each spot, and a list of proteins identified by mass spectrometry, together with theoretical molecular weights, isoelectric points, accession number and Mascot score

Spot	Protein	*P*- value Litter	*P*-value Time	Accession	Theoretical MW (kDa)/ Pi	Mascot score
Proteins that showed decreased abundance over time in challenged birds						
534	Cytochrome b-c1 complex subunit 1, mitochondrial Alpha-enolase	0.042	0.042	gi|507543754 gi|6048768	53.4/6.58 47.6/6.17	910 641
530	Heat shock cognate 71 kDa protein Heat shock 70 kDa protein	0.041	0.0082	gi|45384370 gi|55742654	71.0/5.47 69.9/5.53	1504 812
30	Protein disulfide-isomerase A3 precursor dihydropyrimidinase-related protein /Collapsin response mediator protein CRMP-62	0.041	0.0057	gi|45383890 gi|3122036	56.5/5.76 62.7/5.96	1272 486
523	Adseverin	0.041	0.0033	gi|347360989	81.1/5.53	519
556	Collagen alpha-2(VI) chain precursor Collagen alpha-1(VI) chain precursor l-caldesmon	0.041	0.0067	gi|45384382 gi|49225581 gi|212243	110.3/5.66 109.0/5.63 59.0/8.66	1038 490 287
513	Transitional endoplasmic reticulum ATPase	0.041	0.0040	gi|113206112	90.0/5.14	261
11	Heat shock protein 70 Heat shock cognate 71 kDa protein	0.043	0.012	gi|30962014 gi|45384370	70.1/5.66 71.0/5.47	599 307
522	Collagen alpha-1(VI) chain precursor	0.039	0.0033	gi|49225581	109.0/5.63	532
24	T-complex protein 1 subunit epsilon	0.046	0.0044	gi|60302774	60.2/5.53	941
88	B-creatine kinase	0.041	0.037	gi|211235	42.5/5.78	937
12	Annexin A6 NADPH--cytochrome P450 reductase	0.041	0.017	gi|50982399 gi|307775405	75.6/5.57 77.3/5.42	1336 528
33	Protein disulfide-isomerase A3 precursor Plastin-3	0.039	0.0033	gi|453838905 gi|7530180	56.5/5.76 71.3/5.51	2873 97
77	Aminoacylase-1-like isoform 1 Protein disulfide-isomerase A3 precursor	0.046	0.0057	gi|363738588 gi|45383890	46.3/5.98 56.5/5.76	442 424
29	Tubulin alpha ATP synthase subunit beta, mitochondrial Protein disulfide-isomerase A3 precursor	0.041	0.0066	gi|223280 gi|71897237 gi|5383890	46.3/5.0 56.7/5.59 56.5/5.76	451 140 135
509	Heat shock protein 108 Endoplasmin precursor	0.041	0.0067	gi|194220334 gi|45383562	91.4/4.98 91.7/4.83	524 523
520	Adenosine deaminase	0.041	0.0044	gi|57529377	41.1/5.82	1131
Proteins that showed increased abundance over time in challenged birds						
133	Smooth muscle gamma actin Beta-actin Actin, alpha skeletal muscle B isoform 2	0.041	0.017	**gi|**2967678 gi|63018 gi|50803534	42.3/5.31 42.1/5.29 42.4/5.16	310 262 247
220	Calcium-binding protein p22	0.041	0.0057	gi|56118996	22.5/4.97	345
40	Histone-binding protein RBBP4	0.041	0.029	gi|45382339	47.9/4.74	56
84	Tropomyosin alpha-1 chain	0.041	0.023	gi|45382323	32.9/4.73	484
102	Desmin	0.041	0.0044	gi|2959450	51.7/5.30	330
552	Desmin Smooth muscle gamma actin Actin, cytoplasmic type 5 ATP synthase subunit beta, mitochondrial precursor Vimentin Heat shock cognate 71 kDa protein	0.041	0.0033	gi|2959450 gi|2967678 gi|56119084 gi|71897237 gi|114326309 gi|45384370	51.7/5.30 42.3/5.31 42.2/5.3 56.7/5.59 53.2/5.09 71.0/5.47	1193 1002 937 218 133 132
502	Apolipoprotein A-I	0.043	0.029	gi|211159	30.7/5.58	1020
550	Alpha-enolase Hypothetical protein CJMB04_24e12 26S protease regulatory subunit7 Beta-enolase	0.046	0.0044	gi|46048768 gi|53135040 gi|57525333 gi|46048765	47.6/6.11 23.8/6.79 49.0/5.72 47.6/7.28	872 555 331 137
546	Actin, aortic smooth muscle	0.041	0.00444	gi|71895043	42.4/5.23	1063
73	Beta-actin Smooth muscle gamma actin	0.046	0.023	gi|63018 gi|2967678	42.1/5.29 42.3/5.31	263 256
Proteins that show variable, yet significant changes in abundance over time in challenged birds						
134	PREDICTED:pyridoxal kinase	0.048	0.0066	gi|27465053	32.8/4.65	2136
126	Alpha-tropomyosin 2	0.046	0.0066	363728772	35.2/5.93	244

**Table 2:** Spots differentially abundant for either time or between challenge and control birds. The p-values for each variable: time and litter are given for each spot, and a list of proteins identified by mass spectrometry, together with theoretical molecular weights, isoelectric points, accession number and Mascot score

Spot	Protein	*P*- value Litter	*P*-value Time	Accession	Theoretical MW (kDa)/ Pi	Mascot score
Spots that showed significant differential abundance over time						
190	Actin cytoplasmic type 5 Actin, aortic smooth muscle	0.079	**0.003**	gi|56119084 gi|71895043	42.2/5.3 42.35.23	629 559
198	Actin, cytoplasmic type 5 Actin, alpha skeletal muscle	0.083	**0.003**	gi|56119084 gi|71894831	42.1/5.3 42.3/5.23	512 471
512	Heat shock protein HSP 90-alphad Heat shock cognate protein HSP 90beta	0.113	**0.006**	gi|157954047 gi|47604960	84.4/5.01 83.7/4.95	268 112
503	Apolipoprotein A-I Alpha-tropomyosin 2	0.161	**0.006**	gi|211159 27465053	30.6/5.58 32.8/4.65	589 168
194	Dermcidin preproprotein [Homo sapiens] actin, cytoplasmic type 5	0.275	**0.007**	gi|16751921 gi|56119084	11.3/6.08 42.1/5.3	108 98
106	Actin, aortic smooth muscle Actin, cytoplasmic type 5 Adenosine deaminase Alpha-enolase	0.260	**0.008**	gi|71895043 gi|56119084 gi|57529377 gi|46048768	42.3/5.23 42.1/5.3 41.0/5.82 47.66.17	610 539 364 113
182	Actin, cytoplasmic type 5 Actin, aortic smooth muscle	0.468	**0.008**	gi|56119084 gi|71895043	42.1/5.3 42.3/5.23	611 446
99	Hypothetical protein RCJMB04_22i13 (succinyl-CoA ligase [ADP-forming] subunit beta, mitochondrial-like Beta-actin	0.211	**0.009**	gi|60099069 gi|63018	50.3/6.92 42.0/5.29	694 287
553	Heat shock protein 70 Heat shock cognate 71 kDa protein	0.120	**0.011**	gi|30962014 gi|45384370	70.0/5.66 71.0/5.47	373 304
242	Actin-related protein 2/3 complex subunit 5	0.126	**0.012**	gi|71896007	16.5/5.43	337
142	PREDICTED: protein SEC13 homolog	0.162	**0.012**	gi|363738753	35.6/5.07	151
46	Desmin Vimentin	0.301	**0.013**	gi|2959450 gi|14326309	51.6/5.30 53.1/5.09	2155 209
555	78 kDa glucose-regulated protein precursor	0.068	**0.013**	gi|45382769	72.0/5.12	1947
153	PREDICTED: inorganic pyrophosphatase Beta-actin Smooth muscle gamma actin	0.160	**0.013**	gi|118092623 gi|63018 gi|2967678	33.1/5.53 42.0/5.29 42.1/5.31	526 205 198
13	Plastin-2 Actin, alpha skeletal muscle	0.300	**0.017**	gi|56605886 gi|71894831	70.2/ 5.16 42.3/5.23	936 190
130	Actin, cytoplasmic type 5 Actin, aortic smooth muscle	0.310	**0.018**	gi|56119084 gi|71895043	42.1/5.3 42.3/5.23	879 816
138	PREDICTED: pyridoxal kinase	0.376	**0.023**	gi|363728772	35.1/5.93	387
128	Actin, cytoplasmic type 5 Actin, aortic smooth muscle	0.300	**0.031**	gi|56119084 gi|71895043	42.1/5.3 42.3/5.23	806 744
557	Tubulin beta-7 chain Tubulin beta-3 chain **T**ubulin beta-4 chain Tubulin beta-2 chain	0.359	**0.036**	gi|45384338 gi|153792017 gi|71896203 gi|52138699	50.0/4.78 50.2/4.78 50.8/4.86 50.3/4.78	614 470 416 399
166	Coatomer subunit delta Annexin A6	0.370	**0.042**	gi|118405190 gi|45382029	57.5/5.8 75.6/5.42	281 163
58	Alpha-enolase Hypothetical protein RCJMB04_24e12	0.440	**0.042**	gi|46048768 gi|53135040	47.6/6.17 23.8/6.97	490 305
146	Beta-actin Smooth muscle gamma actin	0.068	**0.042**	gi|63018 gi|2967678	42.0/5.29 42.2/5.31	230 174
Spots that showed significant differential abundance between challenged and control birds						
525	Villin-1	**0.042**	0.050	gi|45382125	92.8/5.88	87
526	Villin-1	**0.046**	0.065	gi|45382125	92.8/5.88	513
135	Actin, cytoplasmic type 5 PREDICTED: actin, alpha skeletal muscle B isoform 2	**0.049**	0.118	gi|56119084 gi|50803534	42.1/5.3 42.3/5.16	364 300
17	T-complex protein 1 subunit eta	**0.048**	0.239	gi|71895883	60.8/5.96	716
157	Actin, aortic smooth muscle Proliferating cell nuclear antigen	**0.046**	0.330	gi|71895043 gi|45383776	42.3/5.23 29.1/4.6	618 593
104	Adenosine deaminase Actin, aortic smooth muscle	**0.042**	0.358	gi|57529377 gi|71895043	41.0/5.82 42.3/5.23	620 329

### Western blot analysis

**Fig. 7:**
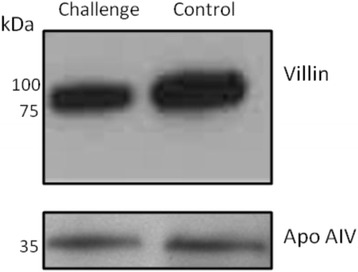
Western blot confirming the decreased abundance of villin in the intestinal lysate of the challenge group at day 22. Image is representative of three western blots with similar results

Western blot analysis of gut lysate from day 22 challenged and control groups reveal different staining intensities for villin-bound antibody (Fig. 7) with lower signal intensity in the challenged birds, validating the DiGE results. Apolipoprotein AIV is synthesised in the intestine and liver, with the proximal intestine being the primarily site of synthesis [21]. This protein was identified but found not to be differentially abundant between the challenged and control birds at any time point and as such used as a loading control.

## Discussion

### The re-used litter affected growth and intestinal microbiota

By day 22 the mean pen weights of the challenge pens were significantly lower than the control pens. Birds were bulk weighed by pen and uneven growth was evident within the challenged groups, indicating that the used litter was having a significant effect on growth. The presence of *C. perfringens*, *Lactobacillus, C. jejuni* and *Eimeria* in the used litter was expected since used poultry litter harbours bacteria that are intestinal in origin [8] and the litter was heavily laden with excreta from the broilers reared on it. *C. jejuni* was present in the ileal digesta of challenged birds at 22 days of age, while the control digesta remained negative for *C. jejuni.* While a number of organisms in the challenge litter may have contributed to the reduced growth, there is increasing evidence that *C. jejuni,* in addition to its role in bacterial food borne illness in humans, is more than a harmless commensal in chickens as birds do mount an immune response to *C. jejuni* infection [22]. The *Eimeria* oocyst count was within the normal range for litter of this age. Oocyst counts in litter and faeces peak between 4 and 5 weeks during the growth of broiler chickens [23] so for the challenged birds at 12 days of age, the introduction of oocysts from the used litter may have been sufficient, in the absence of anti-coccidials or any acquired immunity, to initiate changes in the intestinal proteome. No obvious signs of coccidiosis was evident on post-mortem examination, however coccidial infections induce a variety of pathological and immunological responses that help the host acquire protective immunity, and immunological and non-immunological defences play a role at the intestinal mucosal surface during *Eimeria* invasion [24]. The interaction between *Eimeria* and the intestinal mucosal immune system is a key component in the defence of the chicken to these enteric pathogens [25]. While it has been shown previously, that infecting birds with different *Eimeria* species changes the serum proteomes of infected birds [26], taking a localised proteomic approach utilising coccidial infection models may yield further insight into how the chickens respond to infection.

### Differentially abundant proteins

In birds challenged with used litter, proteins associated with the stress response, apoptosis and the extra-cellular matrix increased in abundance over time. Actin and actin associated proteins were identified in a large number of spots and were found to exhibit variable changes in abundance overtime and between challenge and control birds. Actin, dominating the proteins that were found to increase in abundance over time in the challenge birds, was identified in four of the ten spots and the actin associated protein tropomyosin was identified in a further spot. Conversely the actin associated proteins adservin, T-complex-1 and plastin-3 decreased in abundance over time in challenged birds. Actin was also found to be differentially abundant in multiple spots that increased in abundance over time in all birds in the study (Table 3). Between hatch and 21 days of age increases in villus volume, crypt depth, proliferation of the enterocytes, cellular hypertrophy and increased rate of migration of enterocytes are seen [27, 28] and the growth of the jejunum and ileum continues to increase beyond 14 days [27]. Enterocyte migration from villus crypt to tip is associated with actin remodelling [11] and the increased actin abundance in multiple spots in all the birds over time is likely to be associated with normal growth and as such is a normal developmental finding, associated with normal epithelial cell turnover, migration and differentiation of the enterocytes.

**Table 3:**
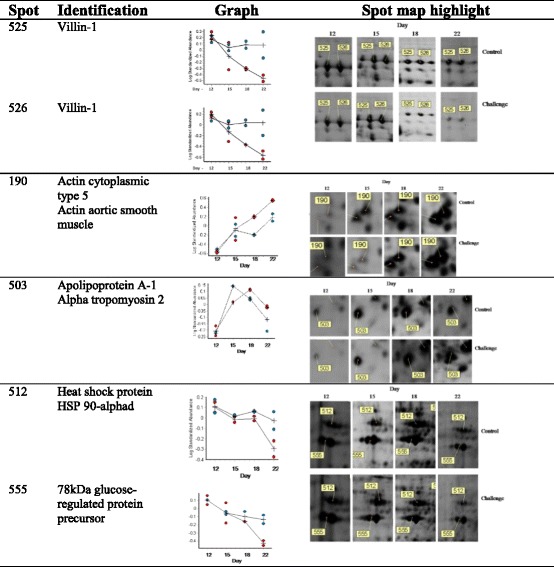
Differentially abundant spots

Villin, the major actin-modifying protein associated with the microvillar actin filaments [29], remained stable over time in the control birds but decreased markedly after day 12 in the challenged birds (Table 3). Localised at the apical surface of the enterocytes, villin regulates epithelial cell morphology, actin reorganisation and cell motility; regulating cell survival by preserving steady state actin dynamics [29, 30]. In the challenged birds the wide spread (over multiple spots) increase of actin, decrease in actin associated proteins, together with notable change in the abundance of villin-1, a key regulator of apoptosis in the GI epithelium [30], makes it highly likely that the microbial challenge increased apoptosis in the intestinal epithelia effecting cytoskeletal remodelling. The cytoskeletal protein tubulin decreased in abundance in the challenged birds whereas desmin and vimentin increased in abundance in the challenged birds over time. As changes in actin and villin are linked to apoptotic cell death [30], the changes in the abundance of these proteins in the intestinal epithelia of the challenged birds may have contributed to the reduced growth rate and changes in protein abundance relative to the control group.

Remodelling of the actin cytoskeleton in response to stress is a fundamental process in eukaryotic cells with a clear link between the actin cytoskeleton and apoptosis established [30]. Apoptosis regulates both the number of stem cells in the crypts as well as the sloughing of cells from the villus tips with expression of villin highest in the apoptosis-resistant villus cells and lowest in the apoptosis-sensitive crypts [31]. A homeostatic balance between proliferation and apoptosis is essential for the intestinal epithelium to function as a physiological and structural barrier [29]. Abnormalities associated with apoptosis in the epithelium have been linked to GI tract diseases in humans [30] where decreased villin production from enterocytes in patients with ulcerative colitis and Crohn’s disease has been related to disturbances in differentiation and maturation processes in the GI epithelium [29]. Serum villin and auto-antibodies specific for villin have been shown to increase in patients with inflammatory GI diseases and colorectal cancers [32, 33], its presence in the serum postulated to be the result of dysplastic cell lysis or the improper processing of villin in tumour cells [33]. While villin is not present in the serum of healthy broilers (O’Reilly, 2015 unpublished observations) its presence in the serum of birds with significant GI pathology has yet to be been determined. Other studies in chickens have identified villin in the intestinal proteome and suggested it to be developmentally regulated, as abundance is highest at day of hatch and declining thereafter up to day 14 [3]. The same study also found villin-1 abundance to differ between broiler lines selected for different growth performance [3].

In evaluating the effect of probiotics on the microbiota and intestinal proteome of broilers, a previous study found *E. faecium* supplementation to affect villin abundance [5]. Of particular note in this previous study [5] is the observation that 7 villin-1 spots were reduced in abundance and a further single villin-1 spot (of lower molecular weight) was increased in abundance [5]. Villin has recently been revealed to function in a pro-apoptotic fashion, whereby its cleavage in the intestinal mucosa produces lower molecular weight pro-apoptotic fragments that sever actin in an unregulated fashion to initiate the extrusion and subsequent apoptosis of effete cells from the villus tips [31]. It is therefore possible to postulate that the observations in this previous study (the reduced abundance of multiple villin-1 spots, and the increased abundance of a single lower molecular weight villin-1 spot [5]) are indicative of villin having both pro- and anti-apoptotic functions in chickens also. Because villin and its associated fragments play such pivotal roles in the maintenance of the intestinal epithelial cell architecture and tissue homeostasis [31], characterisation of the mechanisms by which the intestinal microbiota composition affects their abundance would provide valuable insights into both the pathogenesis of enteric disease and how more subtle changes in diet and microbiota composition affects growth, especially in a challenging environment.

In pigs infections such as those caused by *Salmonella enterica* serovar Thyphimurium increase the abundance of cytoskeletal proteins such as actin in the intestinal proteome, while proteins associated with actin function show variable responses [34], in keeping with the findings in the challenged birds. These authors found villin abundance to decrease in infected pigs and associated this finding with membrane ruffling and microvilli denuding [34]. Colonising germ free pigs with either non-pathogenic *E. coli* or *L. fermentum* resulted in actin and actin proteins associated with remodelling, including tropomyosin, to increase in abundance in the gut tissues of the pigs colonised with *E. coli.* However they were not increased in the gut tissues of pigs colonised by *L. fermentum* [11]. Similar findings have also been reported in mice and zebrafish [35, 36].

Collagen alpha-1 VI, the major collagen type synthesized by intestinal epithelial cells, is involved in the homeostasis of this rapidly renewing epithelium [37]. From day 12, the abundance of this protein decreased in both challenged and control birds, in the challenged birds however the decrease was especially notable; though the abundance did increase again between days 18 and 22, returning to levels closer to that of the control group by day 22. The decrease seen in both groups of birds between days 12 and 15, together with the more dramatic decreased noted in the challenge birds indicates that this protein, a regulator of fibrillogenesis and an anchoring meshwork connecting collagen fibers and other structures such as nerves and blood vessels to the surrounding matrix [37], is highly changeable over this early period of growth. The abundance of collagen alpha-1 VI appears to be influenced by the intestinal microbiota, as evidenced by the significant difference between the challenged and control birds at day 15, 3 days post challenge. It is possible that both the age associated changes in intestinal microbiota seen during this period and the changes induced by the introduction of the used litter could influence the abundance of this protein. Given the critical role collagen alpha-1 VI has in maintaining the integrity and functioning of this highly dynamic epithelium [37] the marked changes in abundance observed as a result of the addition of the used litter is significant.

The abundance of proteins associated with metabolism, energy and TCA cycles and glycolysis and membrane transport reduced in challenge birds over time. Creatine kinase, an enzyme catalyzing the reversible phosphorylation of creatine for cellular maintenance and energy transport [4], decreased over time in the challenged birds; it was also found to be reduced in the intestinal proteome in albusin B supplemented broilers [4]. Cytochrome b-c1, α-enolase and NADPH cytochrome p450 reductase, all enzymes in molecular pathways associated with energy metabolism, such as glycolysis, the electron transport chain and ATP synthesis all decreased in abundance over time in the challenged birds. Annexin-6 also decreased over time in the challenge birds. Previous studies have found annexin proteins to be most abundant in the intestine at hatch and to decline thereafter [3]. Annexin proteins are critical in regulating membrane and membrane-cytoskeleton interactions, having an important role in facilitating exocytoses and stabilisation of membrane domains [3].

Six spots were identified as being differentially abundant in the challenged birds only, with time having no effect. Proliferating cell nuclear antigen and adenosine deaminase were two of these spots. Abundance of proliferating cell nuclear antigen, a protein involved in DNA replication, remained steady in the control birds but increased dramatically in the challenged birds and remained elevated throughout the study period. Adenosine deaminase an enzyme involved in nucleotide metabolism was stable in the control birds but decreased in abundance in the challenged birds after the used litter was introduced at day 12, though abundance increased between days 18 and 22, suggesting a return to pre-challenge levels.

Heat shock proteins (HSP) HSP71, HSP70 and HSP108 decreased in abundance in the challenged birds over time and HSP70, HSP71 and HSP 90 decreased in both groups of birds over time (Table 3). Heat shock proteins are a group of proteins synthesised in response to physical, chemical or biological stresses, including heat exposure [38]. They are molecular chaperones responding to stress related events in a variety of organs including digestive organs. Heat shock proteins protect against environmental stresses and are considered important for adaption to environmental changes [39]. Hyperthermia, mild irritation (gastric acid), secondary ischaemic conditions and oxidative stress caused by lipopolysaccharide (LPS) have been found to induce HSP in the GI tract, though the precise mechanisms *in vivo* are unknown [39, 40]. Intestinal HSP70 in broilers under acute heat stress has previously been found to play an important role in the oxidative stress response [38]. A strong positive correlation between HSP70 abundance and digestive enzyme activity under heat stress has also been identified in previous studies, suggesting that HSP70 may improve intestinal function during acute heat stress [38].

In this study the introduction of the used litter decreased the abundance of all HSP identified in all the birds but especially the challenged group, though the decrease over the four time points did fluctuate between spots. This decrease could be developmentally linked and therefore a normal finding. Some HSPs are constitutively present under basal physiological conditions by specific cells in the body including the intestinal cells [40], as such, decreased HSPs abundance observed in both groups of birds is likely to be part of normal development. Young chicks, immunologically naïve and establishing an intestinal microbiota may constitutively produce HSP in the intestinal tissue. The protective functions HSP, highlighted in studies of heat stress [38, 41] where no changes in intestinal morphology were detected, despite increases in HSP70 [38], indicate that changes at the proteome level are protective. Three heat shock proteins identified in the intestines of broilers treated with *E. faecium* supplement were down regulated suggesting that the intestinal mucosa of *E. faecium* supplemented broilers have reduced inflammatory and oxygen stress and as such require less energy and nutrition leading to improvement in the feed conversion efficiency [5]. Protein disulphide isomerise A3 (PDI A3) was identified in two spots that decreased in abundance over time in the challenge birds. Like HSP, PDI A3 is also involved in protein folding ensuring that proteins adopt their correct folds by catalyzing disulfide bond formation [42] and in both groups of birds there is a dramatic decrease at day 22. This could be because the birds have passed the period of maximal intestinal growth, seen between hatch and 21 days. Beyond 22 days birds enter a phase where intestinal growth slows and this is likely to result in a changes in protein abundance.

Apolipoprotein A-1 (ApoA1) was identified in two spots (502 and 503), both of which showed differential abundance over time (Table 3). Spot 502 significantly increased in abundance in the challenged birds. ApoA1, a major component of high density lipoprotein (HDL) in plasma, is involved in lipid transport and metabolism and plays an important role in cholesterol homeostasis. ApoA1 has anti-inflammatory properties and in mammals is a negative acute phase protein, as plasma concentrations of this protein decrease during acute inflammation [43]. In chickens ApoA1 responds as a negative acute phase protein (O’Reilly, E. L., unpublished observation) as it does in other species [44] reducing its concentration in plasma as a result of inflammation and infection. In avian species ApoA1 is produced in numerous other tissues other than the liver, the primary site of synthesis in mammals [45]. Locally synthesised ApoA1 is thought to act as a local lipid transporter [45] and it is possible that the increase in ApoA1 observed in the challenged birds may have occurred due to an increase in cellular debris, brought about because of the increase in cellular apoptosis.

As a result of a microbial challenge, delivered by the addition of used litter, the intestinal proteome of broiler chickens showed differential abundance of a number of proteins. The results, comparable with other studies of the intestinal proteome of other farm animals and humans indicate that many of the responses to infection and inflammation in the small intestine are conserved among vertebrates, though the decrease in intestinal HSP as a result of microbial challenge and age has not been reflected in other studies. The chicken intestinal proteome appears highly malleable to change, with used litter, supplementation with bacteriocidin [4] and *E.faecium* [5] genetic selection [3] and exposure to atmospheric ammonia [6] all eliciting changes in protein abundance, many of which involve the same molecular pathways. Proteomic studies of the intestine allow a more complete understanding of the interactions that take place at the mucosal-luminal interface, where microbiota, nutrients and pathogens all play a role in determining protein abundance and as such, the growth and health of the bird.

## Conclusions

Using a used litter microbial challenge model in this study resulted in reduced and uneven growth, no mortality and the identification of a large number of jejunal proteins that changed abundance significantly between days 12 and 22 as a result of normal growth and development and also as a result of the microbial challenge. Actin, actin associated proteins and heat shock proteins showed the greatest changes both developmentally and also as a result of the used litter challenge. This study uncovered the effect this type of challenge model has on the intestinal proteome of the broiler chicken, the results of which are highly relevant to many areas of poultry sciences. Identifying and distinguishing the changes in proteins abundance and molecular pathways that occur as a result of normal growth from those that occur as a result of a challenging microbial environment is important in a species able, so effectively, to convert feed into meat protein.

## Abbreviations

ANOVA: Analysis of variance
ASRC: Avian Science and Research Centre
CHAPS: 3-[(3-Cholamidopropyl)dimethylammonio]-1-propanesulfonate hydrate
cm: centimetre
Cy: Cyanine
DiGE: Difference gel electrophoresis
DTT: dithiothreitol
ECL: enhanced chemiluminescence substrate
g: gram
GI: Gastrointestinal
h: Hour
HRP: Horse radish peroxidase
IEF: Isoelectric focusing
IPG: Immobilised pH gradient
k: kilo
kg: kilogram
M: Molar
mg: milligram
mM: milimolar
MOPS: 3-(N-morpholino)propanesulfonic acid
MS: Mass spectrometry
NaCl: Sodium chloride
NCBI: National center for biotechnology information
NCP: Nitrocellulose paper
nLC-ESI-MS/MS: nanoflow HPLC electrospray tandem mass spectrometry
SDS-PAGE: Sodium dodecyl sulfate polyacrylamide gel electrophoresis
SRUC: Scotland’s Rural college
TBS: Tris buffered saline
TBS-T: TBS & tween.
V: Volt
v/v: volume per volume
w/v: weight per volume
Mg: microgram
Ml: microlitre
